# Instructing the Public

**Published:** 1921-04-23

**Authors:** 


### THE PUBLIC WELL-BEING.

### Instructing the Public.

What does the phrase "popular health instruction" mean? As used by the League of Red Cross Societies it refers especially to propaganda among large numbers of the population, including both children and adults, for the purpose of calling their attention to organised health work and of placing them in possession of accurate, desirable information, with the result of creating a public sentiment towards healthy living and better health conditions. There are many countries where a few highly intelligent doctors know exactly—so declares Mr. Edward Stuart, writing in the official organ of the League—what their respective Governments should do to improve conditions, but they are unable to secure the necessary legislation and appropriations, merely because there is no popular demand for improvements, nor is there any general comprehension of what is required. The Serbian town-dweller, for instance, does not know that his typhoid is caused by the water-supply, or he would probably demand a proper installation; the poor mother in a large city does not know what is the proper food for her child, and the child dies when it might be saved were there sufficient demand for welfare centres.

Of course, the kind of instruction necessary depends entirely upon the condition of the country in which it is carried out—the simplest lessons for illiterate peoples; in the more highly developed States concentration on those subjects which are especially pertinent and the presentation of the facts in a form adapted to the education and psychology of the people. The principal methods employed are as follows:—

Bulletin of interest to those already engaged and interested in public health activities.

Pamphlets for children.

Pamphlets for adults.

Posters, drawings, and cartoons to stimulate popular interest.

Instructions in schools by regular courses and by special lectures.

Mass meetings and lectures to the general public.

Moving pictures and lantern slides.

Publicity in newspapers. Exhibits. Novelties.

We shall refer in detail in a subsequent article to the possible scope of most of these methods, only pausing here to point out that under the heading of novelties—which have included in some up-to-date communities Punch-and-Judy shows, clowns, health games, flag-days, aeroplanes dropping circulars, and so forth—nothing but a temporary effect is produced which must not allow attention to be deflected from the main stream of regular and systematic propaganda. Popular health instruction, as Mr. Stuart shows, is undoubtedly on the increase in many countries. In France, stimulated by the extensive propaganda on tuberculosis now being carried on by the Rockefeller Commission, the Ministry of Health is planning to create a division for this work, and the Municipality of Paris has already actively started a permanent campaign along up-to-date lines. The United States Public Health Service and State health departments are increasing their appropriations year by year; Red Cross societies in some countries consider this a part of their peace-time programme; the Serbian Government has appropriated for this year nearly a million francs for this purpose; private organisations such as the Metropolitan Life Insurance Company of New York, the American Social Hygiene Association, the Maternal Tuberculosis Association in New York, the Belgian Anti-tuberculosis League, and many others, are all extensively engaged in this work. There are organisations in Great Britain like the People's League of Health and other voluntary bodies, doing useful propaganda work, the latest manifestations of which are poster and newspaper advertisement types of publicity in regard to venereal disease and the renewed attempts on the part of combined health and social organisations to bring about a reform in the national habits in regard to diet and food generally. At the same time this work properly belongs to the State, and if the Ministry of Health is to achieve its full purpose a continuous publicity campaign against disease and insanitary conditions of all kinds will have to form an important part of its administrative arrangements.

